# HNO Protects the Myocardium against Reperfusion Injury, Inhibiting the mPTP Opening via PKCε Activation

**DOI:** 10.3390/antiox11020382

**Published:** 2022-02-14

**Authors:** Daniele Mancardi, Pasquale Pagliaro, Lisa A. Ridnour, Carlo G. Tocchetti, Katrina Miranda, Magdalena Juhaszova, Steven J. Sollott, David A. Wink, Nazareno Paolocci

**Affiliations:** 1 Department of Clinical and Biological Sciences, University of Torino, 1043 Torino, Italy; 2 Laboratory of Cancer Immunometabolism, National Cancer Institute, NIH, Frederick, MD 20892, USA; ridnourl@mail.nih.gov (L.A.R.); wink@mail.nih.gov (D.A.W.); 3 Cardio-Oncology Unit, Center for Basic and Clinical Immunology (CISI), Interdepartmental Center of Clinical and Translational Sciences (CIRCET), Interdepartmental Hypertension Research Center (CIRI-APA), Department of Translational Medical Sciences, Federico II University, 80131 Napoli, Italy; cgtocchetti@gmail.com; 4 Department of Chemistry, University of Arizona, Tucson, AZ 85721, USA; kmiranda@email.arizona.edu; 5 Laboratory of Cardiovascular Science, National Institute on Aging, NIH, Baltimore, MD 21224, USA; juhaszovam@grc.nia.nih.gov (M.J.); sollotts@grc.nia.nih.gov (S.J.S.); 6 Division of Cardiology, The Johns Medical Institutions, Baltimore, MD 21287, USA; npaoloc1@jhmi.edu; 7 Department of Biomedical Sciences, University of Padova, 35131 Padova, Italy

**Keywords:** myocardial reperfusion injury, nitroxyl (HNO), nitric oxide (NO^.^), PKCε, mitochondrial permeability transition pore (mPTP), K_ATP_ channels

## Abstract

Donors of nitroxyl (HNO), the one electron-reduction product of nitric oxide (NO^.^), positively modulate cardiac contractility/relaxation while limiting ischemia-reperfusion (I/R) injury. The mechanisms underpinning HNO anti-ischemic effects remain poorly understood. Using isolated perfused rat hearts subjected to 30 min global ischemia/1 or 2 h reperfusion, here we tested whether, in analogy to NO^.^, HNO protection requires PKCε translocation to mitochondria and K_ATP_ channels activation. To this end, we compared the benefits afforded by ischemic preconditioning (IPC; 3 cycles of I/R) with those eventually granted by the NO^.^ donor, diethylamine/NO, DEA/NO, and two chemically unrelated HNO donors: Angeli’s salt (AS, a prototypic donor) and isopropylamine/NO (IPA/NO, a new HNO releaser). All donors were given for 19 min before I/R injury. In control I/R hearts (1 h reperfusion), infarct size (IS) measured via tetrazolium salt staining was 66 ± 5.5% of the area at risk. Both AS and IPA/NO were as effective as IPC in reducing IS [30.7 ± 2.2 (AS), 31 ± 2.9 (IPA/NO), and 31 ± 0.8 (IPC), respectively)], whereas DEA/NO was significantly less so (36.2 ± 2.6%, *p* < 0.001 vs. AS, IPA/NO, or IPC). IPA/NO protection was still present after 120 min of reperfusion, and the co-infusion with the PKCε inhibitor (PKCV1-2500 nM) prevented it (IS = 30 ± 0.5 vs. 61 ± 1.8% with IPA/NO alone, *p* < 0.01). Irrespective of the donor, HNO anti-ischemic effects were insensitive to the K_ATP_ channel inhibitor, 5-OH decanoate (5HD, 100 μM), that, in contrast, abrogated DEA/NO protection. Finally, both HNO donors markedly enhanced the mitochondrial permeability transition pore (mPTP) ROS threshold over control levels (≅35–40%), an action again insensitive to 5HD. Our study shows that HNO donors inhibit mPTP opening, thus limiting myocyte loss at reperfusion, a beneficial effect that requires PKCε translocation to the mitochondria but not mitochondrial K^+^ channels activation.

## 1. Introduction

It is now well consolidated that the vast majority of myocardial cell death that concoct the final infarct intervenes during ischemia and the very first few minutes of reperfusion [1]. Finding pharmacological interventions which are able to limit this cell loss after myocardial infarction (MI) would result in better clinical outcomes, i.e., more effective prevention of post-ischemic heart failure (HF).

In cardiac I/R injury, the protective role of gasotransmitters such as nitric oxide (NO^.^) has been investigated for decades [2]. For instance, NO^.^ can maintain coronary blood flow, prevent platelet aggregation, and induce neutrophil-endothelium interaction after I/R injury [3]. Moreover, a low concentration of this gasotransmitter can also increase cardiomyocyte function [4]. However, higher NO^.^ concentrations can achieve quite the opposite effects, i.e., impaired cardiomyocyte function and mitochondrial respiration, as well as enhanced inflammation. In the end, all of these events can lead to worsened post-MI outcomes, owing to exacerbated cell death. Besides dosing and species-related issues, NO^.^ biological actions also depend on its susceptibility to being quenched by reactive oxygen species (ROS), such as superoxide, that can taper its beneficial biological effects [5].

Nitroxyl is the one-electron reduced form of NO^.^ [6]. The chemistry of this reactive nitrogen species (RNS) is significantly different from that NO^.^ and other RNS. For instance, HNO readily reacts with thiol and thiol-containing proteins, and this property is widely used as a tool to explain the possible biological actions of HNO donors [7]. In addition, HNO is relatively inert towards ROS [6]; therefore, its pharmacological benefits can be retained even under highly oxidizing conditions, such as those encountered in the reperfused myocardium [8]. Accordingly, previous work has shown that HNO donors can grant protection against myocardial I/R injury in a manner akin to the early preconditioning effect [9,10,11]. Of relevance, HNO IPC-like effects, but not those of NO^.^ donors, can be quenched by the thiol donating compound N-acetyl-L-cysteine, thus confirming the “thiophilic” nature of HNO biological reactivity [12]. However, the way in which this NO^.^ congener limits I/R injury remains to be deciphered in full.

Protein kinase C (PKC) was the first intracellular kinase shown to be involved in classical ischemic preconditioning [13], and PKCε in particular. Endogenous NO^.^ can trigger a complex signaling transduction pathway involving the activation of PKCε, ultimately leading to the up-regulation of inducible NOS (iNOS) and other cardioprotective proteins (i.e., cyclooxygenase-2 and aldose reductase) to confer resistance against I/R-triggered myocardial damage [14]. Although there are multiple mediators of PKCε activation/translocation, mitochondrial ATP-sensitive K^+^ channels (mito-K_ATP_) appear to be pivotal in this context [15]. Accordingly, activating these channels accounts for IPC beneficial effects, whereas inhibiting them reverses IPC protection [16]. Thus, PKCε activation, the opening of mito-K_ATP_, and the following rise in mitochondrial ROS are crucial events in cardioprotection [17]. Whether HNO follows similar pathways to salvage more myocardium after I/R injury remains to be tested directly.

The mitochondrial permeability transition pore (mPTP) ROS threshold (*t*_mPTP_) reflects the susceptibility of mitochondria to mPTP induction [18,19]. *t*_mPTP_ is the average time required to induce mPTP in targeted mitochondria (usually a row of ~25 adjacent mitochondria). Mitochondria are protected (i.e., desensitized thus more resistant to oxidative stress) if the *t*_mPTP_ time is longer, in contrast to injury-“stressed” or oxidatively damaged mitochondria where the *t*_mPTP_ is shorter. Studies have shown that NO^.^ can inhibit mPTP opening via PKCε activation, also independently from mito-K_ATP_ activity [17]. Whether HNO donors can increase *t*_mPTP_, and if this effect eventually requires PKCε and/or mito-K_ATP_ channel opening.

In the current study, we determined if two chemically unrelated HNO donors—Angeli’s salt and isopropylamine/NO (IPA/NO)—confer protection against I/R injury in isolated rat hearts, comparing HNO donor impact with the protective effects exerted by either IPC or an equimolar amount of a NO^.^ donor [diethylamine/NO (DEA/NO)]. Then, we assessed if HNO donors promote PKCε translocation to the mitochondria and if blocking it with the PKCε translocating inhibitor, PKCV1-2, prevents, in full or in part, HNO anti-ischemic effects. Next, we tested HNO sensitivity to the K_ATP_ channel inhibitor, 5-OH decanoate (5HD). Finally, using a protocol in which oxidative stress is imposed by local ROS photoproduction and achieved by laser photoexcitation of a row of mitochondria within a cell loaded with TMRM, we tested whether HNO donors increase *t*_mPTP_, thus conferring protection against mPTP-induction. Typically, based on the wide variety of drugs tested previously [18], the expected level of mitochondrial protection achieved (as defined above) averages about 35–45% enhancement in resistance to ROS.

## 2. Materials and Methods

Adult rats (Wistar) had at-will access to a standard rodent diet and water, and received care according to the Guide for the Care and Use of Laboratory Animals by the U.S. National Research Council. Animals (*n* = 72; body weight 400–500 g) were thoracotomized after urethane anesthesia (i.p., 1 g/kg) and 10 min after heparin injection (i.m., 2500 U). Hearts were rapidly excised, placed in a saline buffer (ice-cold), and weighed. The isolated hearts were mounted on a Langendorff apparatus endowed with a peristaltic pump for retrograde perfusion with oxygenated Krebs–Henseleit buffer (17.7 mM NaHCO_3_, 127 mM NaCl, 5.1 mM KCl, 1.5 mM CaCl_2_, 1.26 mM MgCl_2_, 11 mM D-glucose, 5 µg/mL lidocaine, and saturated with a 95% O_2_, 5% CO_2_ gas mixture). Hearts were instrumented to monitor coronary perfusion- (CPP) and left ventricular pressure (LVP) by two electromanometers [20]. Perfusion was maintained at constant flow throughout after being set, during stabilization, to achieve a typical CPP (85–90 mm Hg). Drugs were administered using pumps for co-infusion (Terumo, Japan), connected to the inlet valve with a 10% (compared to coronary flow) infusion rate; syringes of the co-infusion pump(s) contained stock solution (10×) drugs. This adjustment guarantees to minimize the effect of extra-flow on mechanical parameters in the isolated and perfused heart setup. A small perforation on the apex of the left ventricle avoided the LV chamber’s congestion due to thebesian flow. Hearts were kept in a water-jacketed chamber (37 °C) and electrically stimulated (~300 bpm). The CPP and LVP signals were digitalized and recorded for off-line analysis performed with LabView software (National Instruments, USA). Data processing with custom LabView applications allowed for quantification of the maximum rate of increase and decrease in LVP during the cardiac cycle. Briefly, contractility was accessed through the first continuous derivative of LVP over time (msec, sampling was performed at 1 Kilohertz). The derivative was then analyzed for maximums and minimums quantify inotropism (dP/dt_max_) and lusitropism (dP/dt_min_) index (For baseline parameters see Table 1).

### 2.1. Experimental Protocols

**Figure 1:**
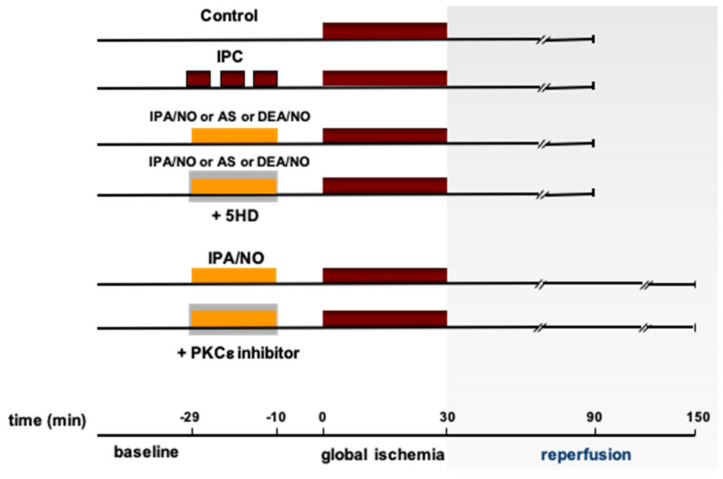
Experimental protocols. All hearts were allowed to stabilize for 30′, after which they were assigned to one of the following experimental condition: Control—no treatment, 30′ global ischemia, 1 h reperfusion; IPC, 3–5′ cycles of preconditioning ischemia, 30′ global ischemia, 1 h reperfusion; Donors (IPA/NO, AS, DEA/NO)—treatment for 19′, 10’ washout, 30′ global ischemia, 1 h reperfusion; Donors + 5-HD, treatment for 19′ with donor and 5-HD, 10′ washout—30′ global ischemia, 1 h reperfusion; IPA/NO—treatment for 19′, 10′ washout, 30′ global ischemia, 2 h reperfusion; IPA/NO + PKCε inhibitor—treatment for 19′ with IPA/NO + inhibitor, 10′ washout, 30′ global ischemia, 2 h reperfusion.

All hearts were monitored during a stabilization period of 20 min after which baseline values were recorded. After stabilization, ex vivo preparations were assigned to one of the experimental protocols described below. All experimental procedures (drug treatment or IP) were followed by 30 min of flow interruption and reperfusion for 60 min (I/R) (Figure 1). Electrical stimulation was stopped during the simulated ischemia and restarted after the third minute of reperfusion. Within the first 10 min of the stabilization time, the coronary flow was modulated to reach the desired CPP and kept constant for the entire experiment (8 ± 2 mL/min*g wet weight). Hearts of the control group (CTRL, *n* = 6) were perfused with regular buffer and did not undergo any treatment after stabilization (see Figure 1). The Ischemic Preconditioning group (*n* = 6, IPC): after stabilization, the hearts were exposed to a preconditioning protocol consisting of three cycles of 3 min of ischemia, separated by 5 min reperfusion and then followed by a 10 min washout with buffer. After the stabilization of the pharmacological preconditioning groups, hearts were treated for 19 min with drugs (single or in combination), followed by a 10 min washout. At the end of washout, hearts underwent a 30 min no-flow ischemia, achieved with peristaltic pump discontinuation, followed by 60 min reperfusion using Angeli’s salt (*n* = 6, AS). 100 mM AS stock solutions were prepared in 10 mM NaOH, as previously recommended [21]. Then, an aliquot was diluted in a plastic 60 cc syringe to reach a concentration of 10 µM AS; then, the rate of the micro-infusion pump attached to a Langendorff inflow side arm was set at 1/10 of the actual coronary flow to reach a final concentration in the coronaries of 1 µM AS, as previously described [11]. AS final concentration was commensurate to the low dose used in a previous in vivo study of I/R and to the one used in other conscious preparations [22]. Vehicle-treated hearts were not included in the current study since we already reported that 100 nM NaOH dissolved in Krebs–Henseleit buffer (vehicle) has no impact on both LV function and infarct size, at a baseline or after global ischemia (REFF). AS was administered with or without the co-infusion of the K_ATP_ channel blocker, 5-hydroxydecanoate (100 µ, AS+5HD in Figure 1). In the diethylamine/NO group (*n* = 6, DEA/NO), hearts were administered with 1 μM final concentration of DEA/NO (stock solution also dissolved in NaOH) for 19 min, followed by a 10 min buffer washout period, with or without co-infusion of 5HD (DEA/NO+5HD). In the isopropylamine/NO group (*n* = 6, IPA/NO, stock solution in NaOH), hearts were exposed to 1 μM final concentration of IPA/NO for 19 min, followed by a 10 min buffer washout period, with or without co-infusion of 5HD (IPA/NO+5HD). Moreover, since IPA/NO has never been tested as a preconditioning agent in rat hearts before, more prolonged reperfusion was applied as follows: “IPA/NO 2 h” group (*n* = 6), after IPA/NO (1 μM final concentration), washout 30 min of ischemia, reperfusion was extended to 120 min. In the IPA/NO+PKC inhibitor (*n* = 6), IPA/NO (1 μM) was co-infused with a selective blocker (PKC v1-2, 10 µM) for 19 min, followed by a 10 min washout, 30 min ischemia, and 120 min reperfusion.

### 2.2. Assessment of Myocardial Injury

IPC has been reported to reduce 50% lactate dehydrogenase (LDH) dismission in isolated rodent hearts after 30 min of ischemia [11]. Samples of coronary effluent (~2 mL) were collected through a catheter placed in the right ventricle via the pulmonary semilunar valve. Sampling was performed 3 min before ischemia, at 3, 6, 10, 20, and 30 min of reperfusion, and every 20 min thereafter. LDH activity was accessed through an enzymatic assay as previously described [11]. Data report cumulative values for the entire reperfusion period (60 or 120 min).

Independent operators assessed infarct area: at the end of the experiment, each heart was rapidly dismounted from the Langendorff infusion, and the LV was circumferentially cut into ~2 mm thick slices. After 15 min of 0.1% nitro-blue tetrazolium solution incubation (37 °C), necrotic tissue was dissected from blue-stained tissue. Necrotic and non-necrotic components were then weighted, and the necrotic tissue volume was expressed as a percentage of total left ventricular mass (area at risk) [11].

### 2.3. Western Blotting

Samples of myocardial tissue were digested in a lysis buffer containing protease inhibitors cocktails (RIPA buffer, Cell Signaling Technology, Danvers, MA, USA). Membrane and cytosolic fractions were obtained through differential centrifugation [23]. Western blotting was performed following standard protocols with equal amounts of protein loaded for each sample (typically 30–60 μg). Protein concentration was measured using the Bradford protein assay (Bio-Rad Laboratories, Berkeley, CA, USA), based on the dye-binding procedure with serum albumin (bovine) used as standard. Proteins were separated by sodium dodecyl sulfate-polyacrylamide-gel electrophoresis (4–12% SDS-Page gradient gels, Invitrogen). After electrophoresis, proteins embedded in the gel were transferred to a PVDF membrane by electroelution. The transfer of proteins was evaluated with a pre-stained molecular weight marker (Biorad Laboratories, Berkeley, CA, USA). After transfer, membranes were saturated with non-fat dry milk and incubated with an antibody cross-reacting to the protein(s) of interest. A horseradish peroxidase-conjugated specific secondary antibody was used to detect luminescence with ECL (Bio-Rad Laboratories, Berkeley, CA, USA). Light intensity was acquired with a Kodak Image Station 440 CF, and densitometry was performed (ImageJ) to quantify levels of expression normalized over actin for cytosolic fraction and Na^+^/K^+^-ATPase for membrane-bound PKCε (primary and secondary antibodies were purchased from Cell Signaling—Na^+^/K^+^-ATPase 3010, PKCε 2683, Actin 4968—and diluted according to manufacturer’s instruction).

### 2.4. Immunohistochemistry Preparation

Samples of the left ventricle were fixed overnight in paraformaldehyde 4%, rinsed with PBS and Glycine (0.2%, twice for 30 min), and cryo-preserved with increasing concentration of sucrose in PBS (7.5%, 15%, and 30% *w*/*v*). A final step with PBS 30% sucrose and OCT (50% *v*/*v*) was performed before directional inclusion in OCT. Next, 7 μm slices were obtained using a cryostat and incubated with specific primary antibodies. DAPI was used to localize nuclei. PKCε was probed with primary antibody (sc-1681, 1:500) and goat anti-mouse IgG secondary antibody (1:10,000, Invitrogen A32727). Slides were analyzed with a Zeiss confocal microscope, and images taken with a 63× magnification lens (oil immersed).

### 2.5. Cells

Adult cardiac myocytes were isolated from Sprague–Dawley rats (2–4 months old) through standard enzymatic techniques [24]. Cells were suspended in a solution containing (in mM): NaCl 137, KCl 4.9, MgSO_4_ 1.2, NaH_2_PO_4_ 1.2, glucose 15, HEPES 20, and CaCl_2_ 1.0 (pH 7.4).

### 2.6. mPTP ROS Threshold (t_mPTP_) Measurements

We have previously developed and extensively tested a model enabling the precise determination of the mPTP sensitivity to oxidant stress in intact cardiac myocytes [18,19,25]. Briefly, small numbers of mitochondria inside isolated cardiomyocytes were exposed in situ to conditions of oxidative stress by repetitive (2 Hz) laser line-scanning (with imaging) of a single row of mitochondria in a cell loaded with 100 nM tetramethylrhodamine methyl ester (TMRM), using a Zeiss LSM 510 inverted confocal microscope (Carl Zeiss Inc., Jena, Germany) with excitation at 543 nm and collecting emission at >560 nm using a Zeiss 63×/1.4N.A.oil immersion objective. This results in incremental, additive exposure of only the laser exposed area to the photodynamic production of ROS and consequent mPTP induction. The ROS-threshold for mPTP induction (*t*_mPTP_) was measured as the average time necessary to induce the mPTP due to the local buildup of ROS in a targeted row consisting of ~25 mitochondria. MetaMorph image analysis software (Molecular Devices Corp., Downingtown, PA, USA) was used to calculate *t*_mPTP_. The data are mean ± SEM.

### 2.7. Chemicals

The HNO donor Sodium trioxodinitrate (Na_2_N_2_O_3_, Angeli’s salt) was generously provided by Dr. Jon M. Fukuto (University of California, Los Angeles, CA, USA). As previously described, stock solutions (100 mM in 10 mM NaOH) of Angeli’s salt were freshly diluted in a buffer prior to use [11]. All other chemicals were purchased from Sigma (St. Louis, MO, USA). IPA/NO was synthetized in the laboratory of Dr. Katrina Miranda.

### 2.8. Statistical Analysis

All values are presented as means ± SEM. All data were subjected to *t*-tests for paired and unpaired data. Significance was accepted at a *p* level of <0.05 (**) and *p* < 0.001 (*). Statistical analysis and graphing of data have been performed with GraphPad Prism.

## 3. Results

### 3.1. The HNO Donor, IPA/NO Limits Infarct Size and LV Dysfunction after I/R in Isolated Rat Hearts

**Figure 2:**
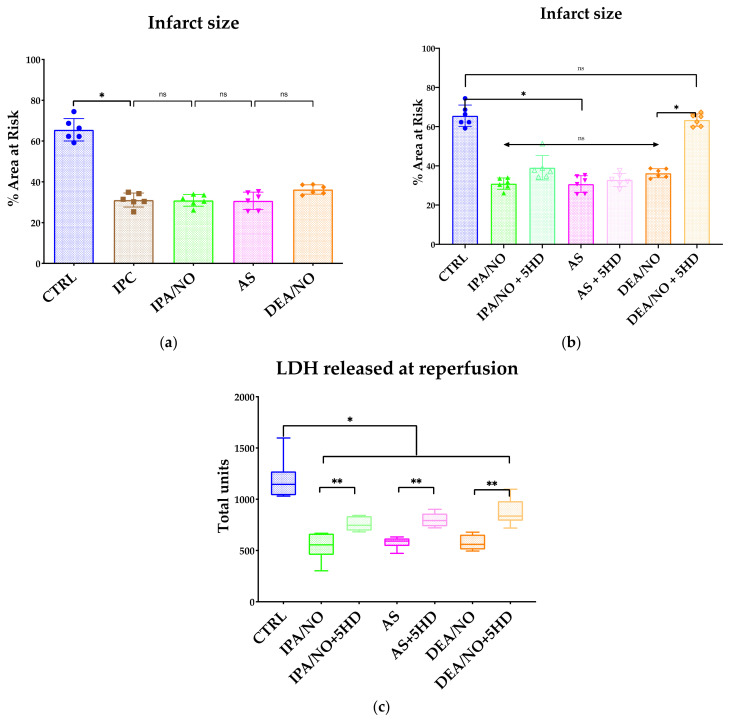
(**a**) Infarct size expressed as percentage of the area at risk (left ventricle mass). Donors (IPA/NO, AS, and DEA/NO) are compared to the classical ischemic preconditioning (IPC). All donors are confirmed to grant a substantial protection against the I/R injury provoked by a 30′ ischemia and 1 h of reperfusion. Data are expressed as Average ± Standard Deviation. * *p* < 0.001, ns: non-significant. (**b**) Infarct size expressed as a percentage of the area at risk (left ventricle mass). IPC and donors (IPA/NO, AS, and DEA/NO) show a significant (*p* < 0.0001) reduction in infarct size compared to the control group, while the use of 5HD differentially affects AS and IPA/NO compared to DEA/NO^.^ No significant differences are observed among the sole donors. *n* = 6 for each experimental group. Data are expressed as Average ± Standard Deviation. * *p* < 0.001, ns: non-significant. (**c**) Myocardial damage expressed by LDH release during reperfusion. Perfusate samples were collected at 0′, 10′, 20′, 30′, 40′, 50′, and 1 h after restarting perfusion. LDH was quantified for each sample and summed. *n* = 6 for each experimental group. Data are expressed as Average ± Standard Deviation. * *p* < 0.001, ** *p* < 0.05, ns = *p* > 0.05.

HNO donated by 10 μM AS administered for 19 min before ischemia markedly reduced both infarct size and myocardial LDH release [11]. However, AS is not a pure HNO donor; it co-releases HNO and nitrite [26]. Hence, to validate that HNO donors can protect the myocardium against I/R injury, we infused an equivalent dose of IPA/NO as a pretreatment to avoid possible confounding effects from nitrite release. At a physiological pH (7.35–7.45), indeed, this primary amine-based diazeniumdiolate behaves as an almost pure HNO donor [27]. In keeping with previous findings obtained with AS [11], IPA/NO also conferred early IPC-like protection when given for 19 min (1 µM) before 30 min ischemia and 1 h reperfusion. In detail, it markedly limited the infarct size expressed as the ratio necrotic-mass/LV-mass, compared to no treatment (30.95 ± 3 vs. 65.53 ± 5.5% (control I/R, *p* < 0.001, Figure 2a). In addition, IPA/NO protective effects were not quantitatively different from that observed with the prototypic HNO donor, AS (Figure 2a). IPA/NO’s beneficial effect (−53% reduction in LV tissue necrosis) was also similar in magnitude to that observed after three IPC cycles (31.08 ± 3.4%, *p* < 0.001 vs. CTRL; NS vs. IPA/NO). In unison with these findings, LDH release was also significantly abated after IPA/NO: from 1342 ± 82 U/mg (control I/R) to 534 ± 82 (IPA/NO I/R; *p* < 0.001 vs. control I/R) (Figure 2b). Again, this amelioration was not different from that afforded by IPC cycles (424 ± 41 U/mg; *p* < 0.001 vs. control; *p* = NS vs. IPA/NO I/R group), nor was it different from that granted by equimolar amounts of the NO^.^ donor, DEA/NO (Figure 2a). From a functional point of view, at reperfusion, LV contractile function was compromised in all groups. However, recovery from myocardial stunning observed in the control I/R group was significantly improved by IPA/NO^.^ Accordingly, post-I/R injury LVDEP was 81 ± 9 mmHg (IPA/NO I/R) vs. 22 ± 4 mmHg (control I/R group), while post-I/R injury dP/dt_max_ was 1688 ± 119 (IPA/NO I/R) vs. 344 ± 180 mmHg/s (control I/R group). IPA/NO functional recovery was not dissimilar from that observed after IPC cycles: LVDEP was 103 ± 20 mmHg (IPC I/R) vs. 22 ± 4 mmHg (control I/R group), and dP/dt_max_ was 1658 ± 540 (IPC I/R) vs. 344 ± 180 mmHg/s (control I/R group, *p* < 0.001). Similarly, DEA/NO impact on post I/R LV function was superimposable to that found in all other (pharmacological or IPC) treatments. In-keeping with the infarct size findings, the total LDH release after I/R injury was not different in the I/R hearts treated with either HNO or NO^.^ donors, or IPC (Figure 2c). Of note, 19 min of continuous infusion of HNO or NO^.^ did not significantly alter basal LV function parameters (data not shown).

### 3.2. IPA/NO Affords Long-Lasting Protection after I/R Injury

**Figure 3:**
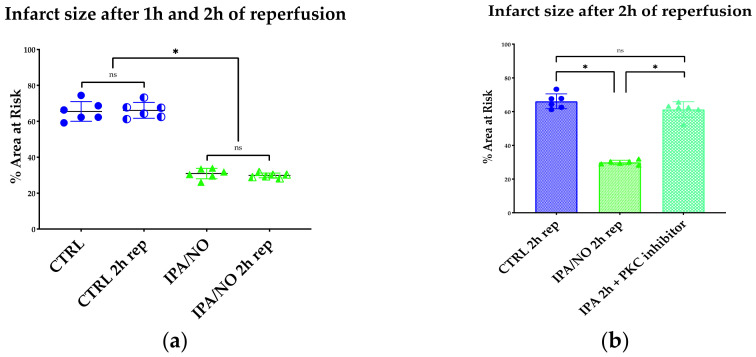
(**a**) Infarct size expressed as a percentage of the area at risk (left ventricle mass). Neither the CTRL nor IPA/NO-treated heart showed a significant difference in terms of necrosis after 2 h of reperfusion when compared to 1 h. *n* = 6 for each experimental group. Data are expressed as Average ± Standard Deviation. * *p* < 0.001, ns: non-significant. (**b**) Infarct size expressed as a percentage of the area at risk (left ventricle mass). IPA/NO protection is abolished by co-infusion of a specific inhibitor of PKC translocation. *N* = 6 for each experimental group. Data are expressed as Average ± Standard Deviation. * *p* < 0.001, ns = *p* > 0.05.

Many studies have used 2 h reperfusion as an additional, translationally meaningful time point [28]. Therefore, in a separate set of studies, we sought to determine whether IPA/NO protection would last up to 2 h reperfusion. We found that, at this additional time point with *n* = 6, the extent of infarct size necrotic tissue volume—expressed as a percentage of total left ventricular mass (area at risk)—was 30.4 ± 3.4%, i.e., not different from that observed in 1 h-reperfused IPA/NO-treated hearts (Figure 3b). In aggregate, this set of data and those reported above suggest that HNO donors are as effective as NO^.^- releasing compounds in granting protection after global I/R in the isolated rat heart, and are durable.

### 3.3. IPA/NO-Afforded Protection Involves PKCɛ Activation and Translocation

**Figure 4:**
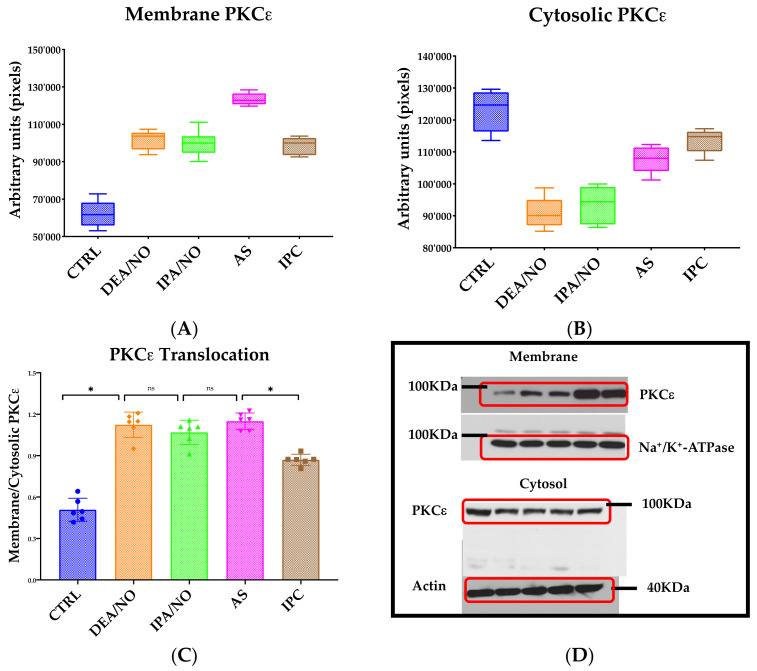
(**A**) Quantification of membrane PKCε in myocardial tissue of ischemic hearts. Isolated hearts perfused with DEA/NO, IPA/NO, AS, or preconditioned with IPC. Membrane PKCε is higher in all treatments when compared to the CTRL group. All readings have been performed in triplicate maintaining the same ROI. (**B**) Quantification of cytosolic PKCε in myocardial tissue of ischemic hearts. Isolated hearts perfused with DEA/NO, IPA/NO, AS, or preconditioned with IPC. Cytosolic PKCε is lower in all treatments when compared to the CTRL group. All readings have been performed in triplicate maintaining the same ROI. (**C**) Ratio of membrane/cytosolic PKCε in myocardial tissue of ischemic hearts. Isolated hearts perfused with DEA/NO, IPA/NO, AS, or preconditioned with IPC. The ratio is significantly increased in all groups compared to the CTRL (*p* < 0.0001). All donors also show a significantly higher PKCε translocation when compared to IPC (* *p* < 0.001, ns = *p* > 0.05). (**D**) Representative images of Western blot membraned incubated with specific antibodies and revealed in chemiluminescence.

**Figure 5:**
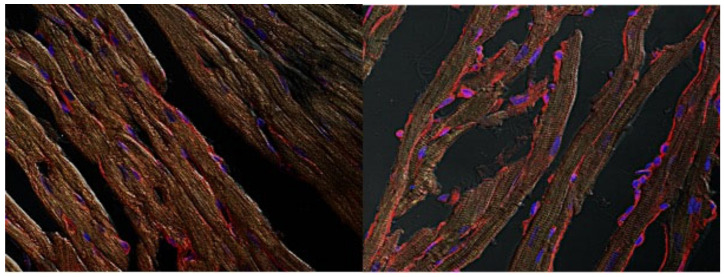
Visualization of PKCε translocation induced by the novel compound IPA/NO^.^ After treatment, hearts were processed to obtain thin slices to be probed with a PKCε antibody. Images were taken by confocal microscopy at 63×. In the left panel, a representative image on untreated, myocardial tissue, in the right panel a IPA/NO treated heart.

NO^.^ beneficial impact against I/R injury is, at least in part, mediated by PKCɛ activation and translocation [29]. Thus, to determine whether, in analogy to NO^.^, PKCɛ activation subserves also HNO cardioprotection, we assessed if IPA/NO pretreatment of I/R hearts would promote the translocation of this soluble kinase to membranes as a prerequisite for its anti-ischemic effect, as previously shown [30]. As depicted in Figure 4C, IPA/NO-treated I/R hearts displayed a marked increase in PKCɛ translocation to myocyte sarcolemma (membrane/cytosolic ratio of 1.07 ± 0.087 A.U.), as opposed to those in the control I/R group, in which this phenomenon occurred significantly less (0.51 ± 0.083, U.A., *p* < 0.001). Ratios of PKCɛ translocation similar to that found in the IPA/NO I/R group were observed after pretreatment with DEA/NO (1.1 ± 0.091) or AS (1.15 ± 0.061, *p* = ns vs. IPA/NO). Conversely, IPC treatment of I/R hearts led to a less prominent membrane PKCε translocation when compared to all other treatments (0.87 ± 0.041, *p* < 0.0001 vs. all treatments). Nevertheless, it was still higher than that found in the control I/R group (*p* < 0.0001). Figure 5 displays representative images of PKCε translocation to myocyte sarcolemma after treating isolated rat myocytes with IPA/NO^.^ These data indicate the equal ability of HNO and NO^.^ donors of translocating PKCε to the sarcolemma, which exceeds that of IPC, at least under the present experimental conditions.

### 3.4. IPA/NO-Conferred Protection Is Not Mediated by Mito-K_ATP_ Channels

The opening of mito-K_ATP_ channels can act as the end effector of the protective signaling cascade initiated by NO^.^ in the setting of cardiac I/R injury [31]. Moreover, previous studies conducted in isolated rat mitochondria have shown that HNO, donated by AS, opens mito-K_ATP_ channels in a manner sensitive to the blockers 5-hydroxydecanoate (5HD) and glyburide [10]. On these grounds, we felt it mandatory to test whether, similar to NO^.^, HNO-induced anti I/R injury action also required the opening of these channels in the isolated rat heart. Figure 2a,b show that DEA/NO-induced anti-ischemic effects were sensitive to the 5HD that nearly abolished NO^.^-induced protection, both in terms of infarct size and LDH release. In stark contrast, this K_ATP_ channel blocker did not significantly blunt the infarct size-sparing effects evoked by both HNO donors, i.e., AS and IPA/NO^.^ Of note, however, 5HD significantly reduced the extent to which both AS and IPA/NO suppressed LDH release from the I/R myocardium. This set of data suggests that, differently from equimolar amounts of available NO^.^, HNO anti-ischemic effects are insensitive to mito-K_ATP_ channel blockers, such as 5HD, at least in the isolated rat hearts, and at a dosage shown to block NO^.^-induced cardiac protection [32].

### 3.5. HNO Donors Enhance the mPTP ROS Threshold in Isolated Myocytes

**Figure 6:**
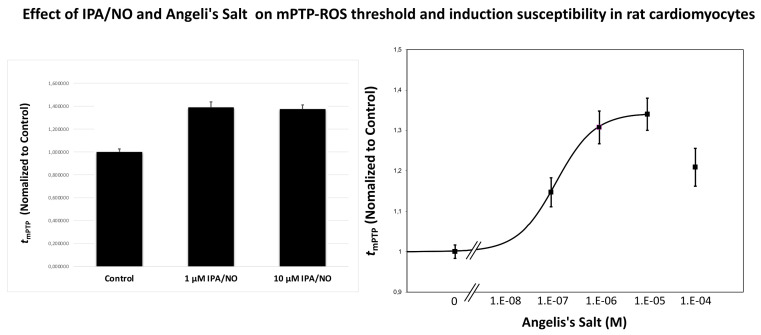
Effect of IPA/NO and Angeli’s Salt on mPTP ROS threshold in isolated cardiac myocytes. Mitochondria loaded with TMRM were laser line-scanned until mPTP induction. The average time required for the standardized photoproduction of ROS to cause mPTP induction (*t*_mPTP_), normalized to that value under Control conditions, is taken as the index of the ROS threshold in that cell. Both, 1 and 10 µM IPA/NO exerted significant protection against the mPTP induction by ROS. Angeli’s Salt enhanced mPTP ROS threshold in a dose-dependent manner with the maximum protection at 10 µM. All experiments were performed at least in triplicate, with cell numbers greater than 12 in each independent experiment. The data are mean ± SEM.

The enhancement of mitochondrial permeability transition pore (mPTP) ROS threshold (*t*_mPTP_) is directly correlated to the degree of mitochondrial and cell protection [19]. NO^.^ has a dual impact on the mPTP opening. High concentrations of this gasotransmitter can open the mPTP via disulfide bond formation or cause other oxidizing effects, likely mediated by complex NO/ROS interactions with the formation of varies RNS; in contrast, low amounts of NO^.^ appear to inhibit it via S-nitrosylation [33]. The extent of and direction in which HNO donors can modify *t*_mPTP_ is currently unknown. We addressed this relevant mechanistic question using freshly isolated cardiomyocytes from adult rats. We were able to report a dose-dependent effect on *t*_mPTP_ with tested HNO donors, showing that both AS and IPA/NO significantly enhanced *t*_mPTP_ over control levels by about 35–40% (Figure 6). This evidence suggests that HNO anti-ischemic actions is, at least in part, due to the ability of this NO^.^ congener to enhance the mPTP ROS threshold, ultimately preventing myocyte cell death.

## 4. Discussion

The mechanisms through which NO^.^ affords cardioprotection have been increasingly elucidated over the last decades. The same cannot be said about the NO^.^ sibling HNO, the IPC-like beneficial action of which [11] awaits further mechanistic explanations. Here, we provide the following new evidence:

- The pure HNO donor IPA/NO confers cardiac protection against I/R injury in a manner quantitatively similar to IPC, NO^.^ donors, such as DEA/NO, and mixed HNO/nitrite releasers, such as AS;
- HNO protection is mediated by PKCε-dependent signaling, but is independent from mito-K_ATP_ channel activation;
- Mitochondrial protection triggered by HNO donors is the result of desensitizing the mPTP to ROS (enhancing its ROS threshold), which, in turn, delays opening of the pore, thus reducing cell damage as a result of ischemia-reperfusion injury.

The biochemistry and pharmacology of HNO and its donors have attracted research attention after the discovery that HNO prodrugs can enhance in vivo cardiac contractility/relaxation in healthy [34] and heart failure (HF) preparations [35]. Recent clinical studies in healthy volunteers have shown that the infusion of newly developed HNO donors is well-tolerated up to 24 h, and associated with a favorable hemodynamic profile [36]. Additional clinical trials (namely, the StandUP-Imaging trial) were conducted in patients with stable HF with reduced ejection fraction (HFrEF), testing the proprietary compound, Cimlanod (Bristol Myers Squibb, New York, USA). This clinical investigation demonstrated that the HNO donor infusion is reasonably well-tolerated compared with a placebo, and that it can reduce congestion markers. Although additional studies are needed to evaluate HNO impact on its actual target population, i.e., acute HF (AHF) [37], all of this evidence supports the pharmacological potential of HNO donors as a treatment for HF, and possibly other cardiac disorders.

In tandem with these promising hemodynamic properties, we and others have already shown that AS-generated HNO, given before a prolonged ischemic event, triggers protective effects in isolated and perfused rat hearts [11]. The present study adds to this scenario two relevant mechanistic clues to HNO anti-ischemic effects: (1) the involvement of PKCε activation, and (2) HNO ability to enhance the mPTP ROS threshold in isolated rat myocytes. Of note, these beneficial effects appear not to be associated with the activation of mito-K_ATP_ channels. In contrast, the latter seem to be required to explain NO^.^-evoked protection in an I/R injury setting, at least to some extent.

Subjecting the heart to one or more brief (3–5 min) ischemic periods with intervening recovery before initiating prolonged ischemia (i.e., IPC) is one of the most effective ways of protecting the heart against I/R injury, along with the use of a variety of pharmacological tools, including volatile anesthetic agents—a procedure termed anesthetic preconditioning [38]. PKC activation is central to IPC phenomenology (see Figure 4). PKC can be activated by factors released during the brief ischemic episodes, such as adenosine, noradrenaline, bradykinin, and endorphins through their receptors, or ROS production at reperfusion. Accordingly, PKC inhibitors and ROS scavengers counter IPC, while adenosine agonists and PKC activators reproduce IPC effects [39]. One intriguing possibility is that, in the presence of O_2_, HNO (donated by either AS or IPA/NO) leads to the production of a radical with an oxidative capacity similar to ONOO^−^ [40,41] that, in turn, oxidatively modifies, i.e., activates PKC. Given the existing literature attesting to the mild oxidizing ability of HNO and its donors [6,42], this eventuality appears very likely, but remains to be tested directly. In the same vein, we do not yet know whether HNO favors the release of additional factors, such as adenosine and bradykinin. Answering these questions requires future, in-depth studies.

Once activated, one possible target of PKCε is mitochondrial ATP-dependent potassium channels, as demonstrated by studies employing openers such as diazoxide, or blockers such as 5DH [39,43]. Previous reports have documented that both NO^.^ and ONOO^−^ can promote the opening of mito-K_ATP_ channels, and that an oxidative burst can induce IPC-like conditions following mito-K_ATP_ channels opening [19,44]. This evidence is consistent with present findings showing that blocking mito-K_ATP_ channels with 5HD prevented the protection afforded by the NO^.^ donor, DEA/NO^.^ Conversely, this blocker did not offset HNO-evoked anti-ischemic effects. This finding highlights the first possible mechanistic divergence between the NO^.^ vs. HNO signaling path towards cardiac protection against I/R injury. At the same time, however, we are aware that a substantial body of experimental evidence has cast some doubts about the specificity of these agents (reviewed in Javador et al., 2003) [39]. They may indeed have off-target effects [45]. Therefore, present evidence documenting apparent independence of HNO protective effects from mito-K_ATP_ channel opening must be validated with alternative approaches, such as using thallium(TI+)-based systems to measure mito-K_ATP_ directly, as conducted in isolated mitochondria [45].

Another possible target of IPC is the mPTP. The threshold for the mitochondrial mPTP induction by ROS (*t*_mPTP_) is significantly reduced after ischemia reperfusion injury, and contributes to cell death [39,46]. We have previously demonstrated that mPTP is the end-effector of protection signaling in the heart and inhibition of mPTP opening with certain pharmacological agents, including the mito-K_ATP_ channel openers, which protect the heart, and that the enhancement of *t*_mPTP_ is directly linked to the level of cell protection [19]. For the first time, our present studies show that cardioprotection induced by HNO donors IPA/NO and AS occurs, at least in part, via reducing mPTP induction by ROS. This evidence begs another critical question: whether HNO (released from its donors) directly inhibits the mPTP opening, or acts more in an in situ IPC-like fashion, i.e., by modulating secondary factors, such as calcium overload and oxidative stress. On the same line of reasoning, Javador and colleagues have shown that IPC prevents initial mPTP opening in rat hearts subjected to 30 ischemia followed by reperfusion, enhancing subsequent pore closure while the heart is recovering [39]. Moreover, these authors demonstrated that the mPTP opening in IPC isolated heart mitochondria occurs more rapidly than in control mitochondria, implying that mPTP inhibition by IPC in situ is secondary to other factors, such as calcium overload and oxidative stress. They went on to show that cyclosporin A—a potent inhibitor of mPTP—rescues the heart from I/R injury better than under control (no treatment) conditions. However, when they evaluated mPTP opening in Langerdorff-perfused rat hearts via 2-deoxy[3H]glucose ([3H]DOG) perfusion (measuring mitochondrial [3H]DOG entrapment), these authors found that agents, such as cyclosporin, are less effective than IPC at preventing mPTP opening. This evidence led them to conclude that countering factors that trigger mPTP opening rather than inhibiting the mPTP directly can achieve better protection against I/R injury. It is very tempting to interpret our present findings in light of the postulation reported above. Indeed, HNO can act both as a mild oxidizer and a Ca^2+^ mobilizer [39]. Therefore, we can speculate that, before direct inhibition of the pore, HNO may leverage on secondary factors, such as levels of calcium and local mitochondrial ROS pools, to delay ROS-induced pore opening, thus attenuating cell death after I/R injury. Testing these intriguing possibilities (direct vs. indirect inhibition of mPTP opening) warrants additional studies.

## 5. Conclusions

Here, we employed a pure HNO donor, IPA/NO, to (1) determine whether HNO is as effective as NO^.^ and IPC in conferring protection against ex-in vivo I/R injury, and (2) to start dissecting the molecular steps explaining how HNO deploys its anti-ischemic effects. Our study suggests that HNO is as effective as classical IPC and NO^.^ releasing compounds in attenuating myocardial I/R injury. Moreover, in analogy with IPC and NO^.^, its anti-ischemic action appears to be dependent on PKCε-dependent signaling yet, in contrast, insensitive to mito-K_ATP_ channel blockade, at least in an isolated rat heart system. Mitochondrial protection triggered by HNO donors is likely the result of desensitizing the mPTP to ROS (enhancing its ROS threshold), which delays the opening of the pore, thus reducing cell damage as a result of I/R injury. The current evidence, coupled to the encouraging hemodynamic profile reported in HF patients, hold some promise for HNO donors to prevent or treat post-ischemic cardiomyopathy.

## Figures and Tables

**Table 1:** Summary of baseline parameters (value ± SD).

	CPP (mmHg)	SIST (mmHg)	EDP (mmHg)	dP/dt Max (mmHg/s)	Heart Weight (g)
**Control**	87 ± 7	99 ± 6	8 ± 2	1.783 ± 289	1.77 ± 0.14
**DEA/NO**	83 ± 6	97 ± 8	7 ± 2	2.020 ± 351	1.70 ± 0.15
**DEA/NO + 5HD**	86 ± 9	95 ± 4	9 ± 2	1.596 ± 246	1.70 ± 0.22
**IPA/NO**	82 ± 6	100 ± 5	9 ± 2	1.545 ± 238	1.67 ± 0.08
**IPA/NO + 5HD**	88 ± 6	100 ± 3	8 ± 3	1.856 ± 374	1.63 ± 0.23
**AS**	85 ± 9	101 ± 4	10 ± 3	1.921 ± 208	1.70 ± 0.10
**AS + 5HD**	82 ± 8	98 ± 10	10 ± 4	1.595 ± 109	1.74 ± 0.15
**IPC**	83 ± 6	99 ± 5	9 ± 6	1.852 ± 298	1.74 ± 0.10

## Data Availability

Data is contained within the article.
